# Ebola Virus IgG Seroprevalence in Southern Mali

**DOI:** 10.3201/eid2706.203510

**Published:** 2021-06

**Authors:** Sidy Bane, Kyle Rosenke, Ousmane Maiga, Friederike Feldmann, Kimberly Meade-White, Julie Callison, David Safronetz, Nafomon Sogoba, Heinz Feldmann

**Affiliations:** University of Sciences, Techniques, and Technologies of Bamako, Bamako, Mali (S. Bane, O. Maiga, N. Sogoba);; National Institutes of Health, Hamilton, Montana, USA (K. Rosenke, F. Feldmann, K. Meade-White, J. Callison, H. Feldmann),; Public Health Agency of Canada, Winnipeg, Manitoba, Canada (D. Safronetz)

**Keywords:** Ebola virus, ebolaviruses, filoviruses, serology, viruses, zoonoses, Mali

## Abstract

Mali had 2 reported introductions of Ebola virus (EBOV) during the 2013–2016 West Africa epidemic. Previously, no evidence for EBOV circulation was reported in Mali. We performed an EBOV serosurvey study in southern Mali. We found low seroprevalence in the population, indicating local exposure to EBOV or closely related ebola viruses.

The West Africa Ebola virus disease (EVD) epidemic of 2013–2016 mainly affected the countries of Guinea, Sierra Leone, and Liberia; its cause was Ebola virus (EBOV; genus *Ebolavirus*, species *Zaire ebolavirus*) strain Makona (*1*). EBOV was introduced into Senegal and more noticeably into Nigeria; it was also exported into several countries in Europe as well as the United States (*1*). Overall, this outbreak was the largest on record, resulting in ≈30,000 EVD cases and 11,000 deaths (*1*). During this epidemic, EBOV was also introduced twice into Mali from Guinea, both times through the border crossing close to Kouremalé (Figure). One introduction came through a young child who had laboratory-confirmed EVD, which resulted in no transmission despite intimate contact with others (*2*). The second introduction came through an imam who had non–laboratory-confirmed probable EVD, with limited transmission. In total, Mali reported 9 cases and 7 deaths throughout 2014 (*2*). Before those introductions, EBOV or other filovirus infections were not previously reported from Mali. As of April 2021, limited efforts have been made to investigate EBOV prevalence in the country, and a small study did not reveal serologic evidence for human exposure to EBOV (*3*).

**Figure:**
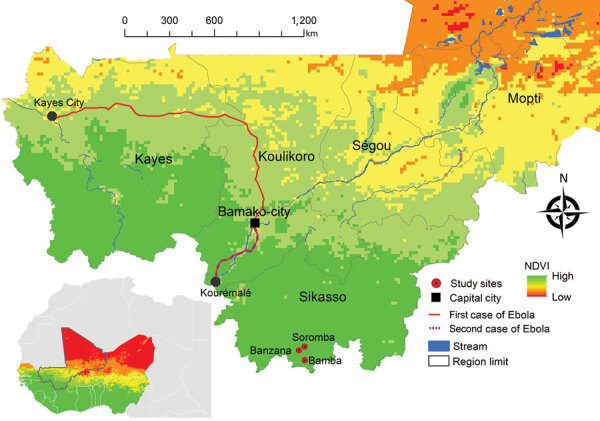
NDIV map showing 3 study sites, Bamba, Banzana, and Soromba (red circles), for investigation of Ebola virus IgG seroprevalence in southern Mali. Red line indicates first Ebola virus introduction into Mali; purple dashed line indicates the second. Inset map shows NDIVs of countries in West Africa. NDIV, normalized difference vegetation index.

Southern Mali borders Cote d’Ivoire, Guinea, and Burkina Faso. This region shares 1 ecosystem; therefore, southern Mali is likely to harbor similar arthropod, rodent, and bat species as the neighboring countries, suggesting the possibility that similar zoonotic pathogens may be present (*4*,*5*) (Figure). Therefore, we tested human serum samples originally collected in southern Mali for Lassa fever surveillance (*6*) for the presence of EBOV antibodies. 

## The Study

 We used 600 serum samples from healthy volunteers collected in 2015 in Bamba, Banzana, and Soromba, located in southern Mali, close to the border with Cote d’Ivoire (Figure) (*6*). The human study protocol was originally approved to determine the seroprevalence for Lassa virus and then later addended to also identify the seroprevalence for EBOV (protocol nos. 15-I-N023 and 18-I-N060). We used 2 commercial ELISA kits (Alpha Diagnostic International, https://www.4adi.com) that detect human IgG to Zaire EBOV nucleoprotein (NP) and glycoprotein (GP). We performed the assays according to the manufacturer’s instructions by using heat-inactivated serum samples (56°C for 60 min). All tests were run in a biosafety class IIa cabinet by personnel wearing personal protective equipment, including N95 face mask, face shield, laboratory coat, and double gloves.

All serum samples were first screened at a 1:100 dilution using the anti-EBOV GP assay. We observed unexpected high reactivity at this serum dilution (122/600; 20.3%) (Table) that was probably unspecific low-affinity binding or cross-reactivity with other viruses. To reduce unspecific reactivity, we next tested all positive serum samples at 1:400 dilution using the anti-EBOV GP assay, resulting in 3.7% (22/600) positivity, and anti-EBOV NP assay, resulting in 4.0% (24/600) seropositivity (Table). Finally, we tested all samples that were positive at 1:400 dilution at a 1:1,600 serum dilution; anti-EBOV GP assay had 0.2% (1/600) positivity and anti-EBOV NP assay 0.7% (4/600) seropositivity (Table). Our testing algorithm considered positives at a 1:100 dilution an equivocal test result. A positive reaction at a serum dilution of >1:400 was considered a positive test result. Using this algorithm, we detected antibodies to EBOV GP, EBOV NP, or both in 37/600 (6.1%) of the study population. Nine (1.5%) participants had positive IgG responses to both EBOV NP and GP antigens (Table). Our results indicate that the population in southern Mali has had or still has exposure to EBOV or closely related ebolaviruses. The overall seroprevalence range was 1.5% (seropositivity in both) –6.1% (a single assay >1:400). 

**Table:** Results of EBOV serology on 600 human samples from southern Mali*

Location	No. (%) positive										
	EBOV GP IgG				EBOV NP IgG				Both		
	1:100	1:400	1:1,600		1:100	1:400	1:1,600		1:100	1:400	1:1,600
Bamba	23 (11.5)	2 (1.0)	0		ND	6 (3.0)	2 (1.0)		NA	1 (0.5)	0
Banzana	39 (19.5)	9 (4.5)	1 (0.5)		ND	5 (2.5)	0		NA	2 (1.0)	0
Soromba	60 (30.0)	11 (5.5)	0		ND	13 (6.5)	2 (1.0)		NA	6 (3.0)	0
Total	122 (20.3)	22 (3.7)	1 (0.2)		ND	24 (4.0)	4 (0.7)		NA	9 (1.5)	0

*A total of 200 samples were tested from each location. EBOV, Ebola virus; GP, glycoprotein. NA, not applicable; ND, not done; NP, nucleoprotein.

Several scenarios may explain the results of this study. First, EBOV or a related filovirus is endemic and circulating in its reservoir species in southern Mali leading to occasional human exposure. This scenario is supported by a similar geographic environment in the southern neighboring countries that had documented EBOV seroprevalence (*4*,*5*) (Figure). A drawback of this hypothesis is the current failure of finding EBOV or closely related viruses in wildlife species, particularly bats, in most West Africa countries. However, Bombali virus, a new *Ebolavirus* species, was discovered in bats in Sierra Leone and Guinea (*7*,*8*); serologic testing has also indicated circulation in pigs in Sierra Leone and Guinea (*9*,*10*).

A second scenario is that exposure in southern Mali was temporary and occurred through human-to-human contact from cross-border movement during the West Africa EVD outbreak. This scenario may be supported by the sample collection time, February 2015 but remains questionable because no patients with EVD symptoms have been reported in this region. However, this also holds true for Lassa virus; 1 case of Lassa fever has been reported in southern Mali despite high prevalence in the local rodent reservoir (*6*).

Third, all seropositivity is due to cross-reactivity with other viruses or to unspecific, low-affinity antibody binding. Filovirus serology, especially for EBOV, has been controversial over the years. Early reports of sometimes high seropositivity in certain regions and populations in Africa were generally thought to be the result of cross-reactivity from the use of assays with low specificity (*11*). However, serologic testing tremendously improved with highly specific and sensitive assays (*12*). Thus, the assumption of false positives as an explanation for the results seems unlikely. The conservation in the NP and GP used as antigens in this study was 67%–75% for NP and 54%–65% for GP among *Ebolavirus* species but does not exclude cross-reactivity among species according to the manufacturer information. In general, GP antibodies are considered more specific due to lower conservation of this protein among ebolavirus species. In our study, the EBOV GP ELISA screening test produced high reactivity at a 1:100 dilution, which probably does not reflect real EBOV seroprevalence as reported from other western and central Africa countries (*13*,*14*). The antigen used in the anti-EBOV GP assay is produced in insect cells; populations may have developed antibodies to insect cell proteins due to exposure through insect consumption (*15*). The NP antigen is produced in *Escherichia coli* bacteria and likely has less protein contaminants than the GP preparation because of more sophisticated antigen purification. However, this concept is speculative; we did not test serum samples in the anti-EBOV NP assay at a 1:100 dilution due to limited sample quantity.

Finally, caution may be necessary when interpreting serologic test results for EBOV and related filoviruses in populations in Africa. Because more reliable, highly specific and sensitive serologic tests are available, more attention should be given to establish proper algorithms for interpretation. Confirmation by independent tests including virus neutralization assays will help. Unfortunately, serum sample volumes in this study were too low to enable such confirmatory testing.

## Conclusions

Given the limitations of our study and a conservative approach for interpretation, our results indicate that the population in southern Mali has been and likely still is exposed to EBOV, other *Ebolavirus* species, or related filoviruses at a seroprevalence of 1.5%–6.1%, which is in the range described previously in west and central African countries (*13*,*14*). Additional work is needed to support this finding, including human surveillance in other regions of Mali. Public health preparedness in Mali should include filoviruses. Initial ecologic studies aiming at identifying potential reservoir species of filoviruses seem justified for southern Mali.
